# Estimating the reproductive number in the presence of spatial heterogeneity of transmission patterns

**DOI:** 10.1186/1476-072X-12-35

**Published:** 2013-07-26

**Authors:** Laura F White, Brett Archer, Marcello Pagano

**Affiliations:** 1 Department of Biostatistics, Boston University School of Public Health, 801 Massachussetts Ave, Boston, MA 02118, USA; 2 NICD-NHLS, Johannesburg, South Africa; 3 Department of Biostatistics, Harvard School of Public Health, 655 Huntington Ave, Boston, MA 02115, USA

**Keywords:** Influenza, Reproductive number, Infectious disease outbreak

## Abstract

**Background:**

Estimates of parameters for disease transmission in large-scale infectious disease outbreaks are often obtained to represent large groups of people, providing an average over a potentially very diverse area. For control measures to be more effective, a measure of the heterogeneity of the parameters is desirable.

**Methods:**

We propose a novel extension of a network-based approach to estimating the reproductive number. With this we can incorporate spatial and/or demographic information through a similarity matrix. We apply this to the 2009 Influenza pandemic in South Africa to understand the spatial variability across provinces. We explore the use of five similarity matrices to illustrate their impact on the subsequent epidemic parameter estimates.

**Results:**

When treating South Africa as a single entity with homogeneous transmission characteristics across the country, the basic reproductive number, R_0_, (and imputation range) is 1.33 (1.31, 1.36). When fitting a new model for each province with no inter-province connections this estimate varies little (1.23-1.37). Using the proposed method with any of the four similarity measures yields an overall R_0_ that varies little across the four new models (1.33 to 1.34). However, when allowed to vary across provinces, the estimated R_0_ is greater than one consistently in only two of the nine provinces, the most densely populated provinces of Gauteng and Western Cape.

**Conclusions:**

Our results suggest that the spatial heterogeneity of influenza transmission was compelling in South Africa during the 2009 pandemic. This variability makes a qualitative difference in our understanding of the epidemic. While the cause of this fluctuation might be partially due to reporting differences, there is substantial evidence to warrant further investigation.

## Introduction

In an emerging outbreak of an infectious disease, such as influenza, there is great interest in determining, amongst other things, its transmissibility, which is typically quantified by its reproductive number. Initially, there is interest in estimation of the idealized basic reproductive number, R_0_, which measures the average number of cases generated by an infected individual in an entirely susceptible population. Post hoc analyses of an outbreak may include estimation of the time-varying effective reproductive number, R_t_, which represents the impact on R_0_ of acquired immunity and public health interventions that typically lead to decreased transmission and a decrease in the growth of the outbreak [1]. Several methods exist for estimating these quantities either in real time as the epidemic progresses, or after the epidemic is over [2,3]. Frequently, these estimators assume a homogenously mixing population, even though often this simplifying assumption may not be realistic. One possible source of heterogeneity is the lack of spatial uniformity in transmission across the population in question. In such an instance, the question arises of how to modify our inference—on the reproductive number, for example—and the impact this might have on our understanding of transmission dynamics.

Typically epidemiological studies of influenza report reproductive numbers over large geographic regions and populations, often estimating a reproductive number for an entire country [4,5]. This may be useful as a metric to compare to previous measures of the same quantity in an effort to determine relative transmissibility of a disease. However, this overall measure confounds information that may impact on transmissibility, or its measure, and it is likely not an insufficiently informative representation of the reproductive number.

Many studies have attempted to measure the spatial dynamics of influenza spread, often in an effort to create better control strategies and predict the occurrence of specific strains in coming influenza seasons [6-8]. Implicit in this work is the reality that influenza transmission dynamics are not spatially uniform, though details of how transmission may vary spatially are lacking.

Spatial considerations are clearly important, as those who live great distances from each other are much less likely to infect one another than those who live in closer proximity to each other. Further, spatial heterogeneity is important to consider since differences may exist in behavioral patterns, demographics, control measures, climate, and other factors that may affect transmission differently in different geographical regions. The issue is made even more complex because reporting issues and healthcare seeking behaviors can vary geographically and also influence data quality. Thus identifying heterogeneity does not necessarily indicate its cause simply.

In this work we introduce a simple modification of the method originally proposed by Wallinga and Teunis [1] to estimate the effective reproductive number. This modification allows for the estimation of the reproductive number(s) in the presence of greater heterogeneity in transmission. We apply this method to data from the 2009 pandemic influenza outbreak in South Africa and estimate the reproductive number for each province. We discuss the potential implications of the results we obtain on future research and surveillance activities.

## Methods

Wallinga and Teunis [1] (denoted WT method hereafter) proposed a network-based method for the estimation of the effective reproductive number by making use of the epidemic curve, **N**={N_1_,…, N_T_}, where N_t_ is the number of cases at time point t, and an estimate of the serial interval, p_1_,…, p_k_, where p_i_ describes the probability of a serial interval of length i and the maximum serial interval length is k. The estimator for R_t_ is a function of the relative probability that case t_i_ was infected by the j^th^ case on day *t′*, denoted $q_{t_{i},{t^{\prime }}_{j}}$ and is given by

Rtj'=∑s=t′+1minT,t′+k∑i=1nsqsi,tj'=∑s=t′+1minT,t′+knsqs,tj',

where n_s_ denotes the number with symptom onset on day s. The relative probability that case t_i_ was infected by the j^th^ case on day $t^{\prime },\;q_{t_{i},{t^{\prime }}_{j}}$, is a function of the probability that case t_i_ was infected by case t_j_′, and is entirely a function of the serial interval, such that P(t_j_′ →t_i_) = $p_{t_{i}-{t^{\prime }}_{j}}$.

### Spatial transmission data

We propose the use of additional structure to describe the probability of an infection event occurring between two cases. We modify the probability of an infectious event between two cases, P(t_j_′ →t_i_), to incorporate spatial information:

$$P\left({t^{\prime }}_{j}\to t_{i}\right)=p_{\left\{t_{i}-{t^{\prime }}_{j}\right\}}d_{t_{i}{}^{\prime }t_{j}},$$

where d_ti′ ,tj_ is a measure of similarity between the cases t_j_′ and t_i_. Note we assume independence between space and the serial interval. By making use of information on conditional probabilities, if known, one could relax this assumption. Since this method only modifies the method of constructing the probabilities that connect individuals in the network, the properties of the estimator originally proposed by Wallinga and Teunis still apply.

This measure can be calculated in a number of ways. The simplest being

$$d_{t_{i}{}^{\prime }t_{j}}=\left\{\begin{array}{l} \;0,\;\mathrm{if}\;\mathrm{the}\;\mathrm{cases}\;\mathrm{are}\;\mathrm{far}\;\mathrm{apart},\\ \;1,\;\mathrm{if}\;\mathrm{the}\;\mathrm{cases}\;\mathrm{are}\;\mathrm{close}\;\mathrm{to}\;\mathrm{each}\;\mathrm{other}. \end{array}\right)$$

The similarity measure can depend on geographical proximity and/or demographic proximity, and can also be informed by observed data on travel or contact patterns. The similarity matrix does not necessarily contain probabilities, but represents the relative similarity between two locations and/or demographic features. Additionally by similarity we are describing the potential for an infective event. Alternatively, we can also entertain a Bayesian formulation and attach a prior to the parameters of the distance measure. Below, we investigate some different measures.

### Data

We use data from South Africa describing the pandemic influenza H1N1 outbreak in 2009. The data contains information on 12,543 reported laboratory confirmed cases, including specimen collection date, gender, age, province of residence, and symptom onset date [9]. There are nine provinces in South Africa that vary substantially in size, population density, climate, and accessibility to healthcare [10]. The symptom onset date was available in 758 cases (6% of the cases). We impute the missing onset dates using a multiple imputation method. To this end we first fit a Poisson model to the lag between onset date and collection date for those who had both dates recorded (715 cases) incorporating statistically significant predictors: (i) the province where the report originated, and (ii) an indicator of weekend versus weekday for the day of collection. The Poisson model was used to randomly generate missing onset times. This process was repeated 500 times creating 500 imputed data sets. All analyses are performed on each of the 500 imputed datasets and results are combined across the individual dataset results and these summaries are the ones we report [11].

Our model requires a similarity matrix. As we are uncertain of which similarity matrix would be most appropriate for influenza in South Africa in 2009, we investigate a variety of matrices in the model and comment on the variability resulting from each similarity matrix. To this end, we investigate five different similarity matrices to describe, what would seem to us to be, plausible transmission patterns between the provinces. The matrices are shown in Tables 1, 2, 3, 4, 5 and are, respectively:

**Table 1:** All transmission occurs within provinces

	***EC***	***FS***	***GT***	***KZN***	***LP***	***MP***	***NC***	***NW***	***WC***
*EC*	1	0	0	0	0	0	0	0	0
*FS*	0	1	0	0	0	0	0	0	0
*GT*	0	0	1	0	0	0	0	0	0
*KZN*	0	0	0	1	0	0	0	0	0
*LP*	0	0	0	0	1	0	0	0	0
*MP*	0	0	0	0	0	1	0	0	0
*NC*	0	0	0	0	0	0	1	0	0
*NW*	0	0	0	0	0	0	0	1	0
*WC*	0	0	0	0	0	0	0	0	1

**Table 2:** Matrix based on reported travel patterns in South Africa

	***EC***	***FS***	***GT***	***KZN***	***LP***	***MP***	***NC***	***NW***	***WC***
*EC*	1.0000	0.0069	0.0479	0.0190	0.0118	0.0095	0.0026	0.0070	0.0293
*FS*	0.0085	1.0000	0.0251	0.0100	0.0062	0.0050	0.0014	0.0036	0.0153
*GT*	0.0248	0.0105	1.0000	0.0291	0.0180	0.0146	0.0040	0.0107	0.0449
*KZN*	0.0240	0.0102	0.0711	1.0000	0.0175	0.0141	0.0039	0.0103	0.0435
*LP*	0.0163	0.0069	0.0483	0.0192	1.0000	0.0096	0.0027	0.0070	0.0296
*MP*	0.0072	0.0031	0.0213	0.0085	0.0052	1.0000	0.0012	0.0031	0.0131
*NC*	0.0034	0.0015	0.0101	0.0040	0.0025	0.0020	1.0000	0.0015	0.0062
*NW*	0.0100	0.0042	0.0296	0.0117	0.0073	0.0059	0.0016	1.0000	0.0181
*WC*	0.0158	0.0067	0.0468	0.0186	0.0115	0.0093	0.0026	0.0068	1.0000

**Table 3:** Uniform probability of transmission between different provinces

	***EC***	***FS***	***GT***	***KZN***	***LP***	***MP***	***NC***	***NW***	***WC***
*EC*	2	1	1	1	1	1	1	1	1
*FS*	1	2	1	1	1	1	1	1	1
*GT*	1	1	2	1	1	1	1	1	1
*KZN*	1	1	1	2	1	1	1	1	1
*LP*	1	1	1	1	2	1	1	1	1
*MP*	1	1	1	1	1	2	1	1	1
*NC*	1	1	1	1	1	1	2	1	1
*NW*	1	1	1	1	1	1	1	2	1
*WC*	1	1	1	1	1	1	1	1	2

**Table 4:** Increased probability of transmission for neighboring provinces

	***EC***	***FS***	***GT***	***KZN***	***LP***	***MP***	***NC***	***NW***	***WC***
*EC*	2	1	0.5	1	0.5	0.5	1	0.5	1
*FS*	1	2	1	1	0.5	1	1	1	0.5
*GT*	0.5	1	2	0.5	1	1	0.5	1	0.5
*KZN*	1	1	0.5	2	0.5	1	0.5	0.5	0.5
*LP*	0.5	0.5	1	0.5	2	1	0.5	1	0.5
*MP*	0.5	1	1	1	1	2	0.5	0.5	0.5
*NC*	1	1	0.5	0.5	0.5	0.5	2	1	1
*NW*	0.5	1	1	0.5	1	0.5	1	2	0.5
*WC*	1	0.5	0.5	0.5	0.5	0.5	1	0.5	2

**Table 5:** Increased transmission for most densely populated provinces

	***EC***	***FS***	***GT***	***KZN***	***LP***	***MP***	***NC***	***NW***	***WC***
*EC*	2	1	0.5	1	0.5	0.5	1	0.5	1
*FS*	1	2	1	1	0.5	1	1	1	0.5
*GT*	0.5	1	2	1.5	1	1	0.5	1	1.5
*KZN*	1	1	1.5	2	0.5	1	0.5	0.5	1.5
*LP*	0.5	0.5	1	0.5	2	1	0.5	1	0.5
*MP*	0.5	1	1	1	1	2	0.5	0.5	0.5
*NC*	1	1	0.5	0.5	0.5	0.5	2	1	1
*NW*	0.5	1	1	0.5	1	0.5	1	2	0.5
*WC*	1	0.5	1.5	1.5	0.5	0.5	1	0.5	2

a. *Diagonal matrix*. This model assumes that all transmissions occur within each province and there is no transmission between individuals in different provinces. This is comparable to applying the original WT method to each province separately.

b. *Travel patterns*. Using data on travel patterns reported by the Department of Environmental Affairs and Tourism in South Africa [12] we construct a second transmission matrix. This one assumes that transmission probabilities mirror the probability of travel between provinces.

c. *Increased transmission between those in the same province*, i.e. d_ij_=2 if i and j are in the same province and d_ij_=1 otherwise. This is an attempt at giving more weight to infection between individuals within a state, but allowing for infection from an individual from another state; a less extreme isolation model than in a., above. Of course, we could entertain values other than 1 and 2 for elements of this matrix.

d. *Neighboring provinces up-weighted*. We define a similarity metric such that if i and j are in the same province the similarity is 2, if they are in neighboring provinces the similarity is 1, otherwise the similarity is 0.5. This model can be thought of as between the model described in a. and the one described in c.

e. *Higher transmission between densely populated provinces*. This matrix allows the three provinces that are the most populous and likely experience the greatest rates of travel to have a greater chance of cross infection. This is done by using the same arrangement as described in c., but allowing the provinces of Gauteng, West Cape, and KwaZulu-Natal to have a similarity measure of 1.5 to each other.

This approach requires an estimate of the serial interval. We make use of the SI distribution between primary cases and suspected plus laboratory-confirmed secondary cases (30%, 17%, 20%, 23%, 7% and 3% for days 1 to 6, respectively) [11].

### Sensitivity analysis

We also perform a sensitivity analysis to assess the robustness of our results to potential errors in the data. Our general approach is to allow the onset date of 10% of the individuals to shift randomly within a 30-day window. We choose one imputed dataset and create fifty “sensitivity” data sets. All analyses are performed on these 50 datasets and compared to the results for the imputed dataset used in the original analysis. Complete results are reported in the appendix.

All analyses were performed in R 2.13.0 (http://www.r-project.org). Programs are available upon request to the corresponding author.

## Results

**Figure 1:**
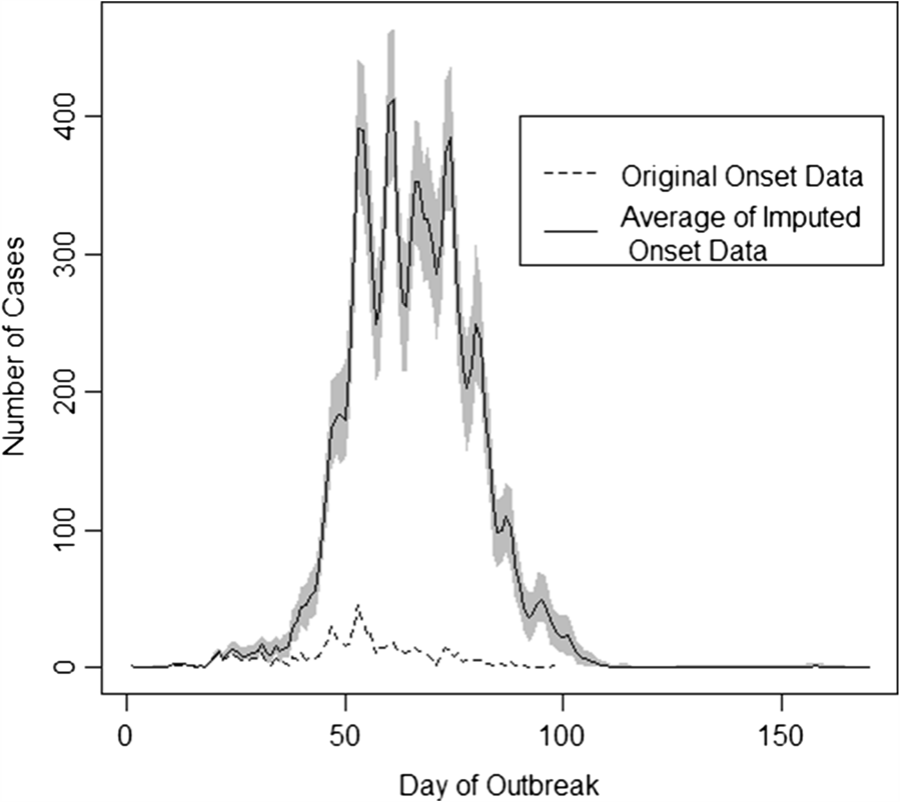
**Imputed epidemic curves.** Gray shading indicates the variability in the imputed data. The dashed line indicates the observed onset data.

**Figure 2:**
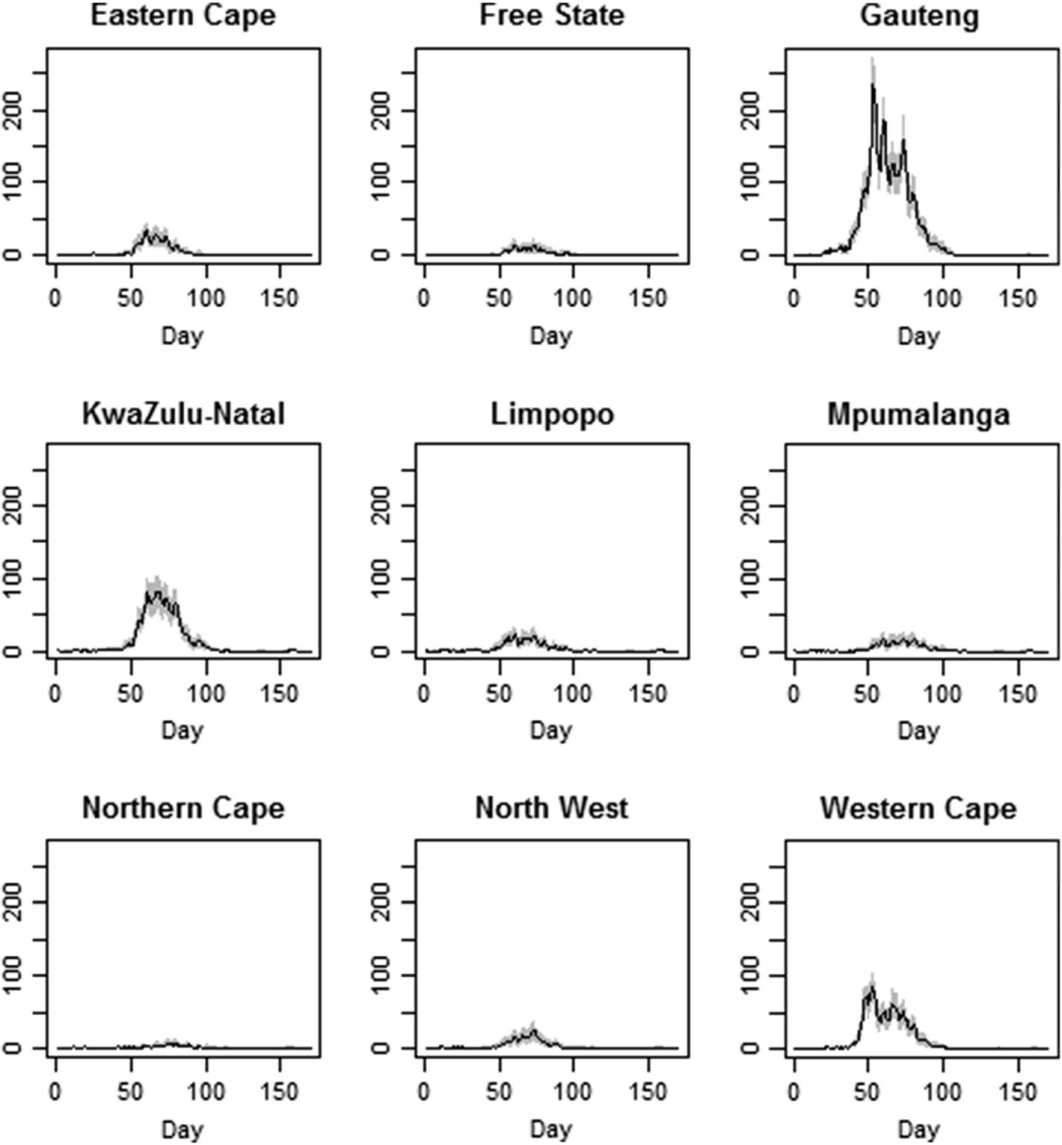
**Epidemic curves for each of the nine provinces.** The shaded area indicates the variability in the imputed values.

Figures 1 and 2 show a sample of the imputed epidemic curves first overall (Figure 1) and then for each of the nine provinces (Figure 2). The first case had symptom onset on June 12, 2009 and the final specimen was collected on November 23, 2009. Gauteng Province had the largest number of cases with 5541 confirmed cases during the epidemic.

**Figure 3:**
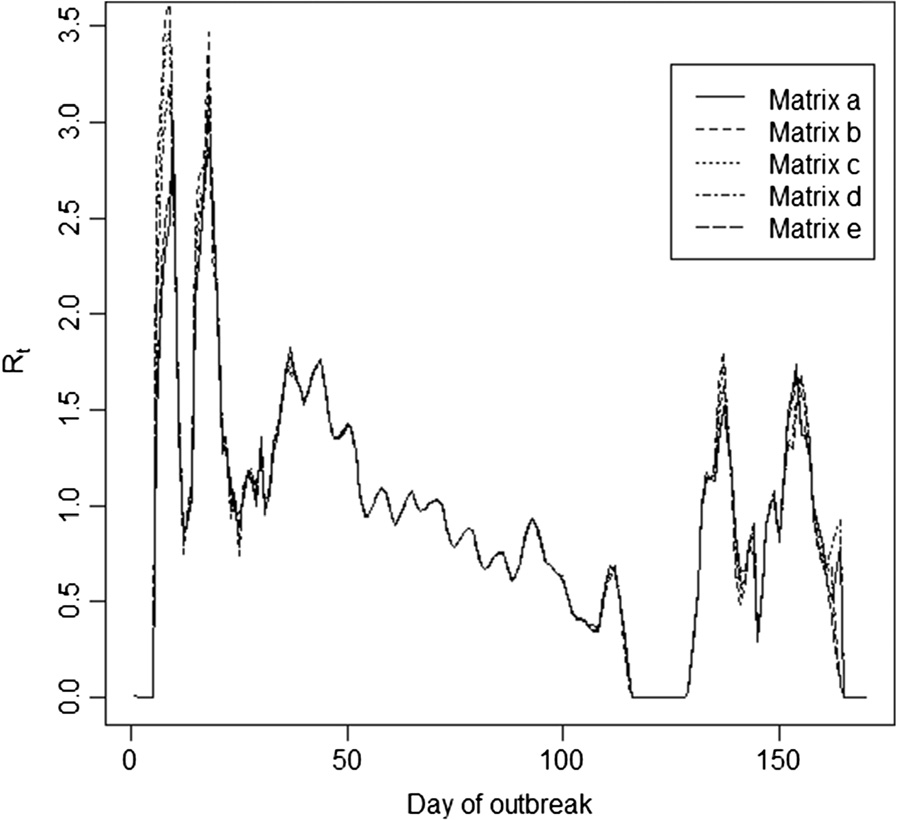
**Estimates of R**_**t **_**using the transmission matrices, as described in the text.** The estimates shown represent the average of the R_t_ estimates obtained across the 500 imputed epidemics. Days when no cases were reported have a R_t_ of 0, though we smooth through this for the purpose of visual presentation.

**Figure 4:**
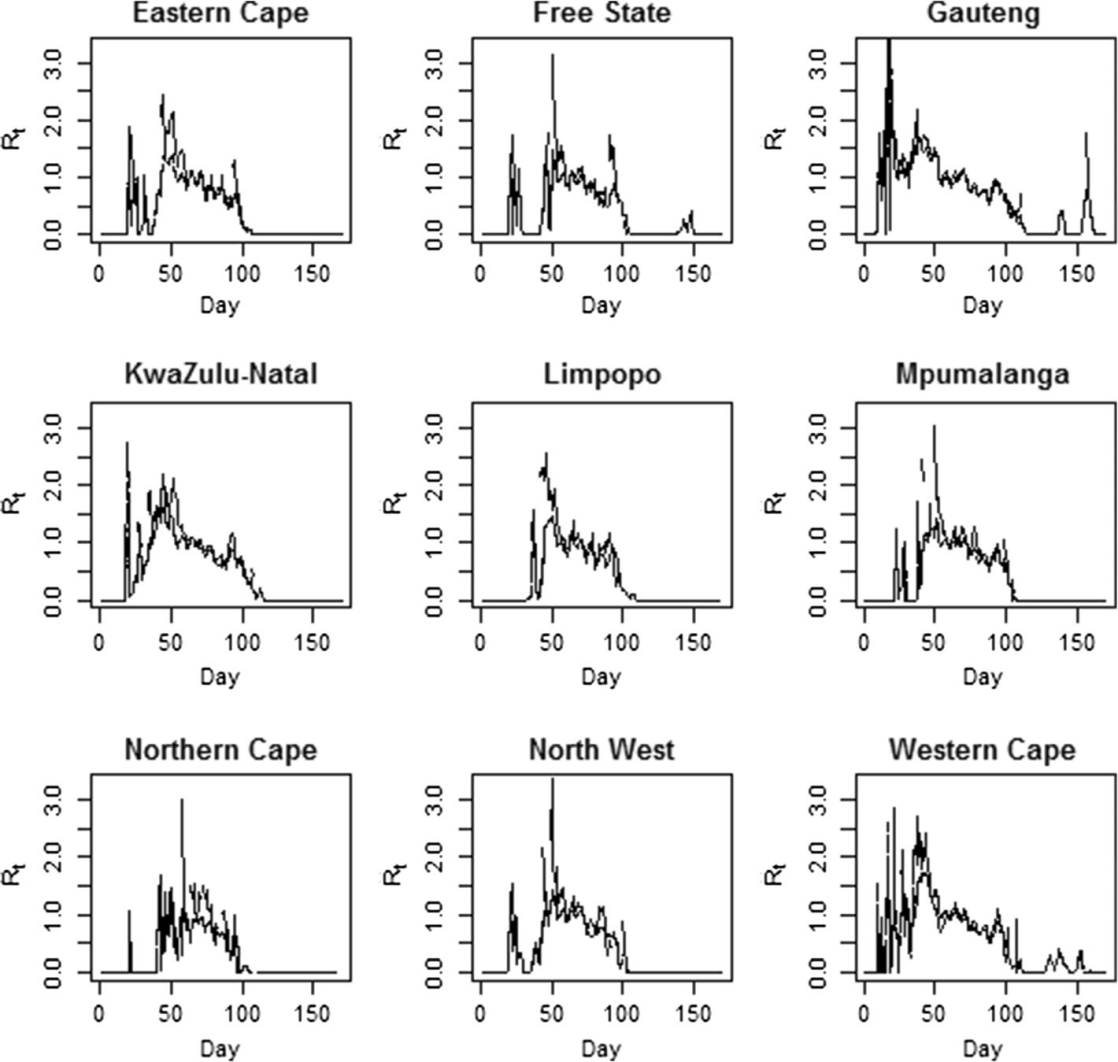
**Estimated R**_**t **_**by province.** The line types for each plot are the same as those used in the previous figure. Days when no cases were reported have a R_t_ of 0, though we smooth through this for the purpose of visual presentation.

Figure 3 shows the overall estimates of R_t_ when the different transmission matrices are used, as well as when the original WT method (Matrix a, ignoring spatial transmission patterns) is used. We note that there are only very minor differences between the estimates. The only noticeable differences exist during the initial and final phases of the epidemic; however, these differences are minimal. Figure 4 shows the results by province, and again there are only minor differences between the estimates obtained with the five different transmission matrices.

Table 6 shows an estimate of R_0_ obtained by averaging the R_t_ estimates obtained over the period of exponential growth in the epidemic (days 10 through 70 reported, though other ranges were used with similar results). We note sizable differences in the estimate of R_0_ between provinces and the transmission matrices used. The biggest differences are between the original WT method (Matrix a) and the methods using a nonhomogeneous transmission matrix (Matrix b-e). Those using a similarity matrix implying heterogeneity are almost identical to each other, but quite different from the value obtained by the original WT method that assumes homogeneity. We note that when nonhomogeneous transmission is assumed, R_0_ is only above 1 for Gauteng and the Western Cape, where Johannesburg and Cape Town are located. One possible explanation is that a certain population density or degree of travel in/out of an area is required to sustain a local epidemic of the flu. The sensitivity analysis yielded results that are consistent with these findings (Table 1).

**Table 6:** **The R**_**0 **_**estimates overall and by region**

**Province**	**a. WT estimate of R**_**0**_	**b. Travel estimate of R**_**0**_	**c. Uniform between provinces**	**d. Extra for neighbor provinces**	**e. Extra for populous provinces**
Overall	1.33	1.34	1.33	1.33	1.34
	(1.31-1.36)	(1.30-1.38)	(1.30-1.37)	(1.30-1.37)	(1.31-1.37)
Eastern Cape	1.33	0.78	0.77	0.94	0.71
	(1.24-1.44)	(0.75-0.82)	(0.75-0.81)	(0.92-0.98)	(0.69-0.74)
Free State	1.32	0.67	0.59	1.00	0.78
	(1.19-1.50)	(0.64-0.71)	(0.57-0.61)	(0.98-1.03)	(0.77-0.81)
Gauteng	1.28	1.51	1.49	1.46	1.42
	(1.27-1.31)	(1.48-1.54)	(1.47-1.53)	(1.44-1.50)	(1.41-1.45)
KwaZulu-Natal	1.37	0.90	0.98	0.95	1.25
	(1.32-1.45)	(0.87-0.93)	(0.96-1.01)	(0.93-0.97)	(1.22-1.28)
Limpopo	1.36	0.68	0.70	0.92	0.72
	(1.18-1.54)	(0.66-0.70)	(0.68-0.71)	(0.89-0.94)	(0.71-0.74)
Mpumalanga	1.31	0.77	0.61	0.98	0.74
	(1.24-1.40)	(0.74-0.81)	(0.59-0.62)	(0.96-1.00)	(0.73-0.75)
Northern Cape	1.23	0.50	0.46	0.82	0.61
	(1.11-1.48)	(0.44-0.55)	(0.45-0.48)	(0.79-0.85)	(0.59-0.63)
Northwest	1.33	0.71	0.65	0.89	0.71
	(1.19-1.50)	(0.67-0.76)	(0.62-0.68)	(0.87-0.93)	(0.69-0.75)
Western Cape	1.34	1.30	1.21	1.09	1.27
	(1.29-1.41)	(1.23-1.35)	(1.16-1.26)	(1.05-1.15)	(1.23-1.32)

The period considered is the 60 days of the outbreak when the epidemic curve was growing exponentially. Results are the median of the results from the imputed datasets with the range from the 500 datasets shown in parenthesis.

**Figure 5:**
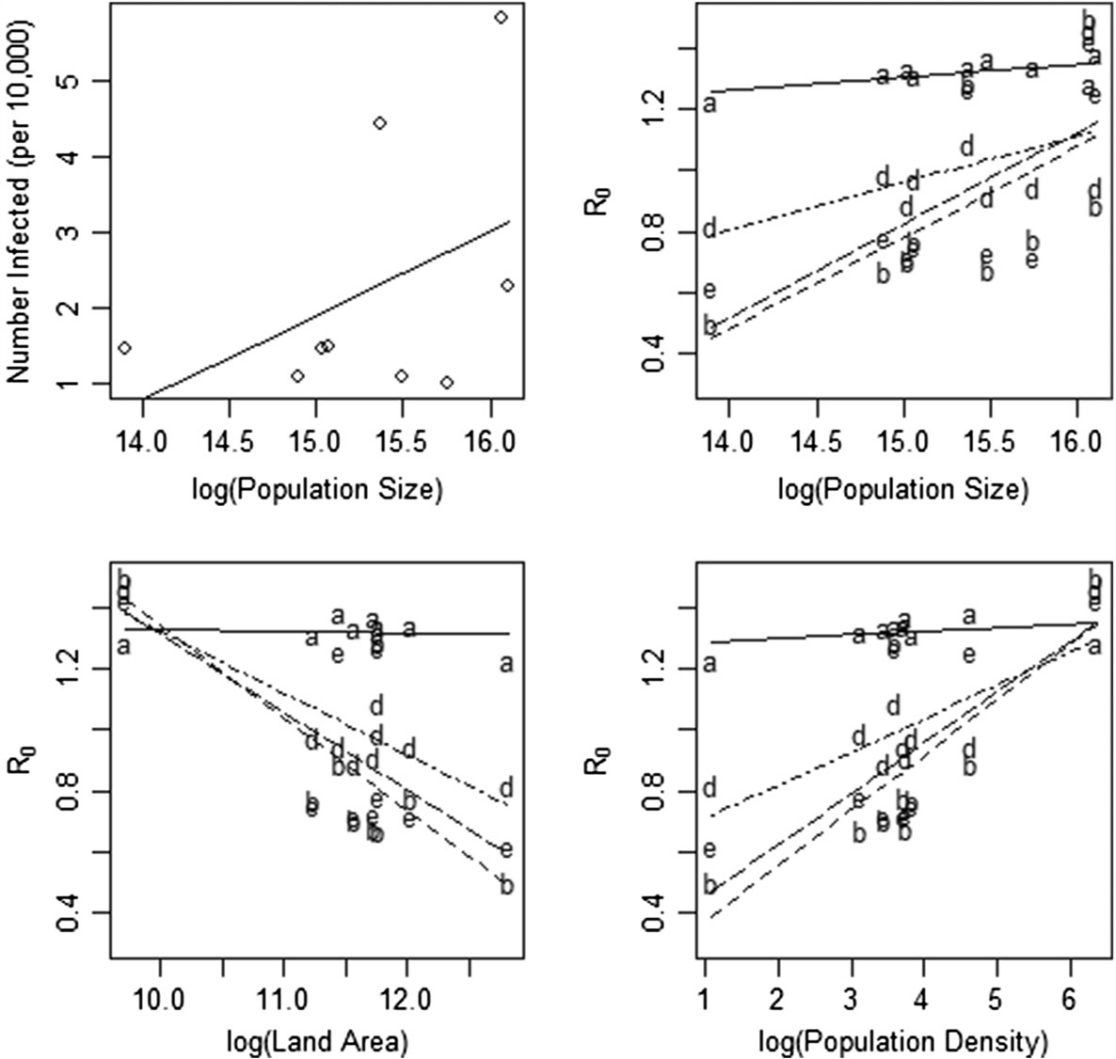
**The relationship between characteristics of each of the provinces and the outbreak.** Lines drawn reflect the least squares regression line for the relationship between the two variables. The first panel shows the relationship between the population size and the size of the outbreak in each province. The second panel describes the relationship between the population size and R_0_. The third panel illustrates the relationship between the land area and R_0_ obtained for each of the transmission matrices. The final panel plots the relationship between population density and R_0_ for each transmission matrix. Line types follow the legend in Figure 3.

We further explore this result in Figure 5, where the estimates of R_0_ are plotted against the population size, land area and population density with the least squares regression estimates shown. The estimate of R_0_ increases with an increase in population size and density (p=0.0004 for matrix b; p=0.04 for matrix c) and decreases with land area. The proportion infected in each province also appears to be related to the population size in the province, though this is not significant (p=0.16). When the WT estimator is used, the model that ignores geography, these relationships disappear (p=0.53 for population density; similar results hold for the other plots), as one might expect.

## Discussion

The results in this paper argue that disease transmission is a function of more than just biology, as is well known, but often ignored. The impact of adjusting the assumption of homogeneous mixing in this South African outbreak, is that apart from the densely populated, urban areas, the pandemic would likely not have been sustained just in the rural, sparsely populated provinces. This finding reinforces the obvious: if individuals have very limited contact with each other, then the outbreaks would probably be small in numbers, limited to small groups, and would likely not propagate to become a larger and more noticeable outbreak. Our estimates of the reproductive number for the more populous provinces are consistent with results reported elsewhere, but the results we obtain for more rural provinces are notably lower [5,13,14].

In our analysis, there are other important issues to consider. Throughout we have assumed that the reporting of cases is uniform throughout the country, and this was the basis for our sizable imputation of the number of symptom onset dates. Even if this reporting is less than 100%, but still spatially uniform, the results we observe will hold [15]. However, if reporting is not uniform between provinces and some provinces have much better reporting of cases than others, we can expect the results to change. For instance, if reporting was lower in the more rural provinces, then it is likely that the estimated reproductive numbers would increase in these provinces if some adjustment for this underreporting were made. Without a more detailed study, this is difficult to quantify and explain. Clearly, there is a certain amount of confounding present, and data reporting issues can be part of an explanation for the results we obtain.

Another factor that can, at least partly, explain the results are the choice of transmission matrices used. We show results for various transmission matrices in order to quantify the degree to which transmission occurs between provinces. Four of these matrices are somewhat arbitrary and not based on actual data. One matrix is based on actual travel patterns in South Africa. But the results are reasonably consistent for the four matrices that assume some degree of transmission between provinces, even when the amount is very small, as in the travel-based matrix. This argues that the results are influenced more by the fact that such a matrix is used and less by the form that such a matrix takes. In all of these cases, despite the substantial differences in the matrices, the result is the same: transmission is maintained in more urban areas and rural areas fail to sustain transmission.

We note dramatic differences between the results when transmission between provinces is incorporated into the estimation (matrices b-e) and the results that assume that no transmission occurs between provinces (matrix a). This reflects the impact of using such matrices and the importance of performing sensitivity analyses to determine the impact of the matrix on the results. Possibly why such matrices have not been used in the past, even though they have a qualitative impact on the results, is that these matrices are difficult to come by, and in practice, they are likely to be estimated in a somewhat ad hoc manner. In some cases, there may be little or no data to inform a transmission matrix. In this case, a wide variety of matrices can be used to determine the plausible range of values that the estimates can assume. Ultimately deriving a method for estimating these matrices, ideally using Bayesian tools, would mitigate this challenge. The framework we provide here lends itself to such an approach, although we have not carried out such an analysis.

We further note the coarseness of the spatial resolution of our data. Our implicit assumption is that individuals within a province are homogenous. While assuming homogeneity within a province is more general than assuming homogeneity over the entire country, it is still a substantial assumption that ignores potentially important variations within a province. As with any analysis, we are limited by the available data, and acknowledge that data on a finer spatial scale would be desirable.

Our method also makes a strong assumption of independence between space and time. That is, we assume that the probability of a particular infector-infectee pair is influenced independently by the temporal and spatial distance between the two individuals. Clearly violations of this assumption are feasible and could impact our results. Without further information on the potential correlations that exist between space and time, any adjustment would be arbitrary and potentially misleading.

While the results we obtain might partially be explained by data quality issues and care-seeking behaviors in rural versus urban populations, there are other potential explanations. A recent cross sectional, serum study reports differential exposure to influenza strains in China across five communities [16], with the most urban community reporting the highest exposure to influenza strains. This provocative result begs further study as it is likely to be attributable to a number of factors and is consistent with the results we have obtained here.

The marked difference in estimates of transmission in rural versus urban areas in our study is also consistent with recent work on social contacts [17,18]. In a study in Japan, there was a significant relationship between the number of social contacts and urbanicity amongst the elderly. Additionally, they similarly found that those in more urban areas have a greater chance of having more supportive interactions [17]. Influenza transmission requires proximity between individuals and social contacts could be one surrogate measure of this proximity.

Additionally, there has been an observed influence of climate and relative humidity on influenza transmission [19-22]. The climate across South Africa is variable with some of the more rural provinces being characterized by a drier climate; the country’s climate is mostly semi-arid, but subtropical along the east coast. So this is not an ideal country to test the transmission theory, but KwaZulu-Natal is the only relatively humid province, so it does not appear that the humidity hypothesis is borne out by these data.

Travel patterns have been correlated with the movement of influenza on a large scale [6,7]. Indeed*,* Viboud et al show that an outbreak that starts in a rural area will spread slowly until it reaches an urban center, at which point it will spread much more quickly [6]. We attempt to incorporate travel patterns in South Africa in our analysis. Travel between the more rural provinces and other areas is much more limited and in general individuals tend to travel to larger provinces, rather than individuals in more urban provinces coming to rural provinces. Thus, the lack of movement between these provinces and areas where transmission is occurring could lead to later onset of sustained transmission locally and lower levels of transmission in the absence of more individuals entering the province and interacting with the local population.

Another explanation is that what we see here could be similar to that observed in the Netherlands in the early phases of the pandemic where the reproductive number was estimated to be below one, indicating that sustained transmission was not occurring and the cases were being generated through imported infections [4]. In South Africa, this would imply that individuals traveled to larger urban centers and became infected there. Their case was reported upon returning home so that the case is not attributed to the location where the transmission event actually took place, at least for the initial cases. We did not account for the possibility of this taking place.

There has been significant work pointing to the great spatial heterogeneity that exists in influenza; however little work has been done to directly estimate the influence of local transmission in propagating this trend. Intensive network models have the capability of investigating these dynamics, but are challenging to implement without extensive resources. Our study introduces a novel and simple approach for doing this by making use of the epidemic curve, information on the serial interval distribution and some prior knowledge of transmission dynamics. We have shown results for estimation over the entire outbreak period, but the modification proposed by Cauchemez et al [2] allowing for real-time estimation of R_t_ could be implemented straightforwardly with this modification, as well.

Our results are suggestive of substantial spatial heterogeneity in transmission dynamics, however further study is warranted due to the limitations of the data at hand and uncertainties on reporting dynamics. At a minimum, these results should argue for modifications in the data that is collected from surveillance and other data collection systems to better understand reporting patterns and the dynamics of interaction between individuals that would lead to substantial heterogeneity in transmission. An improved understanding of heterogeneity will aid in targeting limited interventions in the most effective way possible.

## Competing interests

The authors declare that they have no competing interest.

## Authors’ contributions

LFW and MP conceived the study and developed the methods used. LFW performed the analyses and wrote the manuscript. BA collected the data. All authors reviewed the manuscript. All authors read and approved the final manuscript.
